# The introduction of surgical simulation on three-dimensional-printed models in the cardiac surgery curriculum: an experimental project

**DOI:** 10.2459/JCM.0000000000001577

**Published:** 2023-12-22

**Authors:** Claudia Cattapan, Alvise Guariento, Francesco Bertelli, Francesco Galliotto, Carlotta Vazzoler, Paolo Magagna, Gino Gerosa, Vladimiro Vida

**Affiliations:** a Pediatric and Congenital Cardiac Surgery Unit, Department of Cardiac, Thoracic and Vascular Sciences, University of Padua, Padua; b Cardiac Surgery Unit, Dipartimento Strutturale Cardio-vascolare, San Bortolo Hospital, Vicenza; c Cardiac Surgery Unit, Department of Cardiac, Thoracic, Vascular Sciences, and Public Health, University of Padua, Padua, Italy

**Keywords:** three-dimensional printed models, cardiac surgery, surgical simulation

## Abstract

**Aims:**

Training in congenital cardiac surgery has become more and more difficult because of the reduced opportunities for trainees in the operating room and the high patient anatomical variability. The aim of this study was to perform a pilot evaluation of surgical simulation on a simple 3D-printed heart model in training of young surgeons and its potential inclusion in the curriculum of residency programs.

**Methods:**

A group of 11 residents performed a surgical correction of aortic coarctation using a 3D-printed surgical model. After teaching the surgical procedure, a simulation was performed twice, at different times, and was evaluated quantitatively and qualitatively by a senior surgeon. A 3D model-based training program was then developed and incorporated into our cardiac surgery training program.

**Results:**

A significant improvement in surgical technique was observed between the first and second surgical simulations: median of 65% [interquartile range (IQR) = 61–70%] vs. 83% (IQR = 82–91%, *P* < 0.001). The median time required to run the simulation was significantly shorter during the second simulation: 39 min (IQR = 33–40) vs. 45 min (IQR = 37–48; *P* = 0.02). Regarding the simulation program, a basic and an advanced program were developed, including a total of 40 different simulated procedures divided into 12 sessions.

**Conclusion:**

Surgical simulation using 3D-printing technology can be an extremely valuable tool to improve surgical training in congenital heart disease. Our pilot study can represent the first step towards the creation of an integrated training system on 3D-printed models of congenital and acquired heart diseases in other Italian residency programs.

## Introduction

Training in cardiac surgery has always been challenging because of the limited possibilities for trainee surgeons to practice in the operating room, the high specialization required, and the high risk to patient safety.^1^ Congenital heart surgeons may require even more training, primarily because of the rarity of the disorders and the high variability between defects and patients with the same disease.^2,3^

In recent years, 3D cardiac segmentation and reconstruction of the patient's heart have emerged as interesting innovative tools. This technology is mainly based on the utilization and analysis of standard 2D cross-sectional imaging [such as computerized tomography (CT) scan or cardiac MRI], which are used to reconstruct a 3D model.

3D-reconstructed cardiac models of healthy or diseased hearts are already used for educational purposes with students, nursing staff, and residents.^4–7^ 3D anatomy and the spatial relationship between cardiac structures have particular value for the understanding of congenital cardiac malformations.^8,9^

In the last 5 years, 3D models have played an increasingly important role in our center in the surgical planning for treating complex congenital heart disease or to anticipate a minimally invasive approach in simple disease.^10^ All models have been printed using a transparent plastic resin, which allows the manipulation of cardiac structures and analysis of the external and internal anatomical features of each individual cardiac anatomy. More recently, the introduction of a new elastic resin material has enabled the production of 3D-printed hearts that can be manipulated, cut, and sutured.^11^ This led to the simulation of surgical maneuvers that can be reproduced prior to the surgical procedure that will be performed in the operating room.^12^

Based on this experience, we decided to introduce the use of surgical simulation in the training curriculum of the cardiac surgery residents of our center. Our goal was to allow young surgeons to practice cardiac procedures in a safe environment as often as they wish, and to improve their knowledge and skills.

The aim of this study was to evaluate the effectiveness of surgical simulation on 3D-printed heart models by validating the surgical simulation on a pilot model of aortic coarctectomy performed by our residents. The second goal was to plan a consistent and reproducible simulation training program to be incorporated into our residency program. If proven to be effective, this could then be proposed as part of the curriculum of other Italian cardiac surgery training programs.

## Methods

### The cardiac surgical simulator

In 2021, a surgical simulator called ‘TrainHeart’ (deposit number IT102021000031058) was developed and patented under the aegis of the University of Padua with the aim of reproducing the operating field and the position of the surgeon and the different surgical accesses for the treatment of congenital and acquired heart disease.

**Fig. 1:**
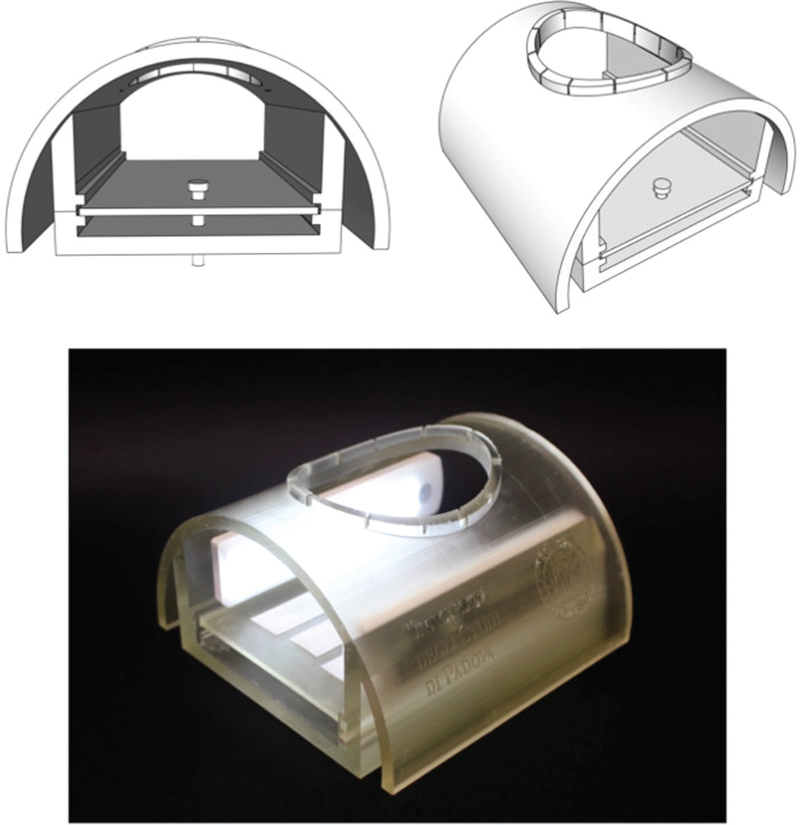
3D-printed surgical simulator (TrainHeart): development of the 3D project (top); final product with integrated LED lights inside (bottom).

The surgical simulator was designed using Shapr3D (Siemens, Parasolid, Budapest, Hungary). This is composed of a static item and a mobile plate. The first part is a semicircular structure with an opening in the upper part to reproduce a sternotomy access (5 × 7 cm). The plate is used to insert the 3D-printed models into the simulator. A row of LED lights is placed inside the simulator on both sides where the heart is positioned to shed light on the operating field. All the elements that compose the simulator were 3D printed using the Sisma Everes Uno 3D printer (SISMA, Vicenza, VI, Italy) and solid resins (SISMA) (Fig. 1). To reproduce different surgical approaches of minimally invasive accesses, we have also developed three different types of covers (for mini-sternotomy inferior/superior, right lateral thoracotomy and left lateral thoracotomy, all with an opening of 3 × 5 cm). These covers were printed in elastic resin (SISMA) using the same printer as mentioned above, thus allowing the cover to be inserted on top of our simulator (Figure S1).

### Validation of the program

**Fig. 2:**
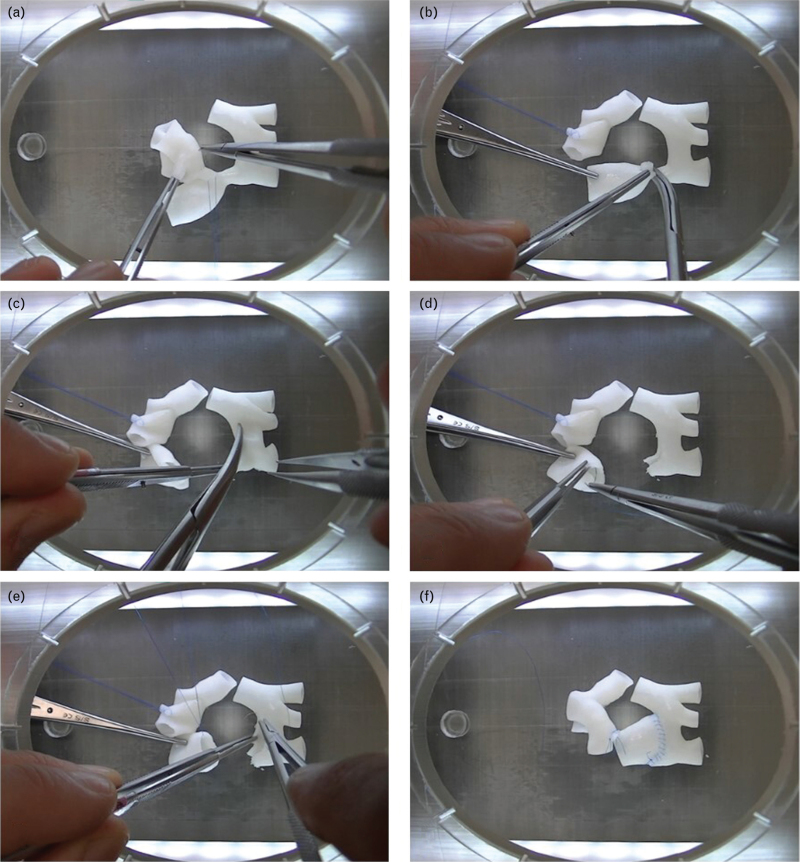
Simulation of surgical correction of an aortic coarctation (coarctectomy) with end-to-end anastomosis on a 3D-printed model. (a) Patent ductus arteriosus ligation; (b) resection of the coarctation; (c) incision of the aortic arch; (d) incision of the descending aorta; (e) suture of the posterior wall; (f) completion of the anastomosis on the anterior wall.

**Fig. 3:**
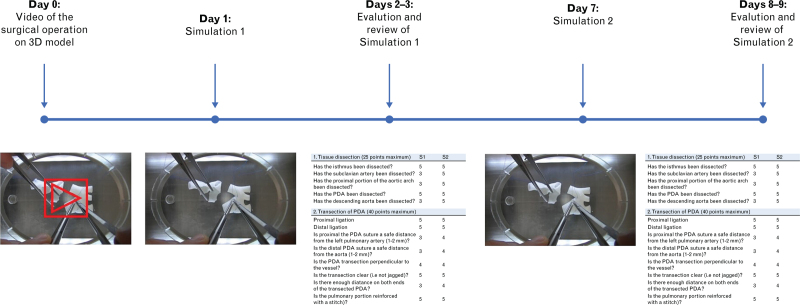
Flowchart of the study.

A total of 11 cardiac surgery residents (including 3 residents in the first year of surgical training, 4 in the second, and 4 in the third year) were enrolled in the study. Residents were provided with a video showing the surgical correction of the aortic coarctation on the 3D-printed model performed by a cardiac surgeon with over 20 years of experience (V.V.) (Fig. 2, Video 1). After giving them 24 h to watch the video and familiarize themselves with the procedure, they were asked to perform the procedure on the model while recording and timing their performance. The video was then reviewed by the staff surgeon (V.V.) and discussed with the resident, explaining any incorrect procedure. A week later, the residents were asked to repeat the surgery simulation again on the 3D-printed model. Again, the recording was examined by the staff surgeon and then reviewed with each resident (Fig. 3).

### Three-dimensional heart models for surgical simulation

Over the last few years, a library of cardiac images from CT, MRI and 2D ultrasound of patients with congenital and acquired heart malformations was created, covering almost the entire spectrum of congenital cardiac malformations.

The models were obtained from a CT acquisition of patients undergoing surgical correction at our institution. They were then reconstructed using the Mimics InPrint software (Materialise, Leuven, Belgium), which allowed an STL model to be obtained. Each model was then modified using Meshmixer (Autodesk Inc, San Rafael, California, USA), thus reproducing the patient's heart. Finally, the 3D models were printed using the Sisma Everes Uno 3D printer (SISMA) and a specific elastic resin (SISMA), which allows flexible models to be obtained. The cost of every single 3D model differs according to the size but is approximately 30 euros.

### Training program development

After validating the feasibility and effectiveness of simulation in the 3D-printed model of aortic coarctation, we sought to validate other 3D-printed models to establish a consistent simulation training process. The final goal was to incorporate it into our residency program and create a more efficient system for our young trainees.^13^

The training program included a monthly simulation session (with a mean duration of 3 h), and the participation was extended to all cardiac surgery residents in our center. To host the various training sessions, a room dedicated to surgical simulation was used, equipped with a large screen for viewing the operation during the simulation and five different stations, all complete with the simulator, a computer station, and video camera to record the procedure.

The simulation program was created and composed of 12 different simulation sessions in order to cover all the basic cardiac surgical procedures and the most common interventions (Table 1). For each procedure, a technical evaluation table was developed with a score to evaluate performance and to monitor the improvement over time. Each resident was provided with a complete simulation kit consisting of a simulator (TrainHeart) completed by the various covers, a set of surgical instruments, various sutures, and the 3D-printed model for the simulation (different each time).

**Table 1:** Surgical simulation training program (University of Padua)

Program A (I–II years)^a^	Program B (III–IV–V years)^a^
(1) Preparation for CPB (part 1) Aortic purse string sutures Aortic cannulation	(1) Revision on the preparation for CPB Aortic purse string sutures and cannulation Right atrial purse string sutures and cannulation Superior vena cava purse string sutures and cannulation Inferior vena cava purse string sutures and cannulation Left ventricle vent purse string sutures and cannulation Purse string sutures for antegrade cardioplegia catheter and placement of the needle-catheter Purse string sutures for retrograde cardioplegia catheter and placement of the cannula
(2) Preparation for CPB (part 2) Right atrial purse string sutures Right atrial cannulation	(2) Vascular anastomosis (conduits with 5 mm of diameter) Termino-terminal Termino-lateral
(3) Preparation for CPB (part 3) Superior vena cava purse string sutures Superior vena cava cannulation	(3) Coronary artery bypass grafting on the heart model Proximal anastomosis on the aorta Distal anastomosis on the left anterior descending branch Distal anastomosis on the posterior descending artery Distal anastomosis on the obtuse marginal branch
(4) Preparation for CPB (part 4) Inferior vena cava purse string sutures Inferior vena cava cannulation	(4) Ascending aorta replacement (prosthetic graft)
(5) Preparation for CPB (part 5) Left ventricle vent purse string sutures Left ventricular vent placement	(5) Aortic valve Aortic valve replacement Annular enlargement
(6) Preparation for CPB (part 6) Purse string sutures for antegrade cardioplegia catheter Antegrade cardioplegia needle-catheter placement	(6) Mitral valve Mitral valve replacement Mitral valve annuloplasty
(7) Preparation for CPB (part 7) Purse string sutures for retrograde cardioplegia catheter Retrograde cardioplegia cannula placement	(7) Atrial septal defect correction with a patch
(8) Approach to right atrium Right atriotomy Right atrium closure	(8) Aortic coarctation repair (end-to-end anastomosis)
(9) Approach to the pulmonary valve Longitudinal MPA/RVOT incision Direct MPA closure Patch MPA/RVOT augmentation	(9) Systemic-to-pulmonary shunt placement Modified Blalock-Taussig Right ventricle to pulmonary artery (Sano modification)
(10) Approach to aortic valve Transverse aortotomy Aortorrhaphy	(10) Supra-valvular aortic stenosis repair (three patches technique)
(11) Vascular anastomosis (conduits with 1 cm of diameter) Termino-terminal Termino-lateral	(11) Transposition of the great arteries correction (arterial switch operation)
(12) Patch positioning with continuous running suture	(12) Norwood operation of hypoplastic left heart syndrome

CPB, cardiopulmonary bypass; MPA, main pulmonary artery; RVOT, right ventricular outflow tract.

a All training sessions were performed on 3D-printed models.

### Design of the resident performance evaluation tools

The surgical simulation of residents was evaluated both quantitatively and qualitatively. The time to complete the correction on the model was first evaluated by comparing the first and second simulations. To carry out a qualitative assessment of the performance, an evaluation table was created by dividing the operation into four parts. Each part included several surgical phases and for each of them, a score from 1 to 5 was assigned according to the accuracy with which it was performed (Table S1). The scores assigned to each step were then added together to obtain a total score, which was expressed as a percentage of a total of 130 points.

### Statistical analysis

The time to perform the surgical simulation and the evaluation of the performance were expressed in terms of median and interquartile range (IQR). A Wilcoxon signed-rank test was performed to compare the score obtained by the residents in two attempts, and the *P*-value for statistical significance was set at 0.05. Statistical analyses were performed using STATA version 15.1 (Stata Corp LLC, College Station, Texas, USA).

## Results

### Validation of the simulation on aortic coarctation three-dimensional printed model

All surgical residents successfully completed the surgical simulations. The time to complete the second simulation was shorter than the initial one (a median time of 39 min, IQR: 33–40 min versus a median time of 45 min, IQR: 37–48 min; *P* = 0.03). The simulation times for each resident are shown in Table 2.

**Table 2:** Qualitative and quantitative evaluation of Simulations 1 and 2

	Score [*n* (%)]		Times (minutes: seconds)	
Participant	Simulation 1	Simulation 2	Simulation 1	Simulation 2
Resident 1	83 (64%)	106 (82%)	46 : 11	37 : 52
Resident 2	84 (65%)	110 (85%)	51 : 16	46 : 54
Resident 3	76 (58%)	108 (83%)	41 : 51	29 : 57
Resident 4	107 (82%)	107 (82%)	41 : 33	39 : 12
Resident 5	85 (65%)	106 (82%)	45 : 04	39 : 06
Resident 6	85 (65%)	118 (91%)	48 : 02	44 : 37
Resident 7	90 (70%)	124 (95%)	35 : 53	33 : 19
Resident 8	79 (61%)	101 (78%)	37 : 03	34 : 09
Resident 9	99 (76%)	125 (96%)	31 : 17	30 : 48
Resident 10	85 (65%)	114 (88%)	46 : 24	42 : 27
Resident 11	66 (51%)	108 (83%)	48 : 30	39 : 47

The quality of surgical performance improved in 10 of 11 residents (91%) (Table 2). The median percentage score from the simulation assessment was higher during the second simulation (83%, IQR: 82–91%) than during the initial simulation (65%, IQR: 61–70%; *P* < 0.001) demonstrating an improvement in technical performance.

### Training program development

A total of 40 procedures were identified and a monthly training program (12 sessions/year) was developed (Table 1). Two different types of programs have been developed: a ‘basic simulation program’ dedicated to residents in their first and second year of training, and an ‘advanced simulation program’ for surgical residents in the remaining 3 years. The ‘basic simulation program’ has been designed to cover basic cardiac surgical techniques in preparation for the ‘advanced program’ where residents can simulate the correction of the most common procedures in both acquired and congenital heart disease.

## Discussion

Training in cardiac surgery and in congenital heart surgery requires enormous technical skills, which are acquired only with repeated procedures.^14^ This is generally not possible in the operating room because of the limited possibilities available to young surgeons. The ability to practice outside the operating room can improve surgical technique and enhance the learning of residents in a cardiac surgery training program.^15^

Surgical simulation has already been shown to improve education in acquired cardiac surgery procedures. Different types of simulators are available, mainly for cardiopulmonary bypass, coronary artery bypass, and mitral valve surgery.^16^ However, these models are usually expensive and dedicated to a single disease or procedure, thus reducing the widespread use of the products.

Due to the high variability of congenital heart disease, tailored models are needed to possibly reproduce all the different anatomies encountered during surgical practice.^17^ For this reason, 3D-printed heart models can be a valuable tool to enrich the training program for congenital heart surgeons.^18^

In this study, we sought to demonstrate how surgical simulation on 3D-printed cardiac models can help improve the surgical skills of young surgeons. The second objective of the study was to establish a consistent and reproducible simulation training program that could be incorporated permanently into our residency program and then potentially exported to other Italian cardiac surgery programs.

First of all, we developed a surgical simulator that was easy to use and with the possibility of recreating different operative accesses. Subsequently, we created an easily accessible library of 3D-printed cardiac models (including both congenital and acquired cardiac anatomies) to be used for surgical simulation and from which we chose an aortic coarctation model for our study.

In this preliminary study, we demonstrated that training in congenital heart surgery using 3D-printed simulation-based training can improve surgical practice, both reducing the time required to perform simulation and increasing the overall performance.

As a consequence, we have developed a training program for cardiac surgery residents to increase exposure to the surgical practice and possibly improve surgical skills. First, a review of the basic surgical techniques was performed. Then, the main acquired and congenital cardiac procedures were analyzed. Our 3D-printed models enable high anatomical fidelity and can reproduce a wide range of disorders and procedures to enhance the surgical practice of young surgeons. The simulation program developed in this study is highly reproducible and with low production costs, allowing large-scale use of the products.

In this study, we demonstrated the possibility of introducing 3D-printing-based surgical simulation in our training program and potentially in other Italian centers. The introduction of surgical simulation as part of the educational curriculum for young cardiac surgeons allows the new generation to practice in a safe environment, review the operation steps as many times as they wish, without risk to the patients, and prepare the trainers for the management of adversity events.^19^

3D printing represents a valuable tool for producing models for cardiac surgery as it allows obtaining different types of congenital heart diseases of different sizes. This would be of great help to young surgeons, allowing them to first practice on a larger model, get comfortable with the technique, and then move on to the smaller model to recreate a child's heart. Finally, 3D-printed models can also be extended to acquired heart disease.

### Limitations

This study has several limitations. First, we ran the evaluation test on a single 3D-printed model and, second, we evaluated the training model on just 11 cardiac surgery residents at a single institution. The ability to add several more 3D-printed models and further evaluations may perhaps help validate and increase the effectiveness of this training opportunity for cardiac surgery residents.

## Conclusion

An effective and reproducible model of surgical training on 3D-printed models was proposed and designed. This was validated on a 3D model of aortic coarctation that was used by a group of residents to reproduce a surgical correction. The trainees showed an improvement in both time and quality. This pilot model can be the first step towards creating an integrated educational system of surgical training on 3D-printed models of congenital and acquired cardiac diseases in other Italian residency programs.

## Acknowledgements

We acknowledge Dr Lucia Zanotto for reviewing the statistical analysis.

Disclosures: The University of Padua and the Azienda Ospedale-Università di Padova share a quote of the patent IT102021000031058 related to the surgical simulator ‘Congenital TrainHeart’.

### Conflicts of interest

There are no conflicts of interest.

## Supplementary Material

Supplemental Digital Content

## Supplementary Material

Supplemental Digital Content
