# Supplementary material for: The introduction of surgical simulation on three-dimensional-printed models in the cardiac surgery curriculum: an experimental project
Source: J Cardiovasc Med (Hagerstown). 2023 Dec 22;25(2):165–72. doi: 10.2459/JCM.0000000000001577 (PMC10836787; doi:10.2459/JCM.0000000000001577)
Supplement: Supplemental Digital Content [file jcarm-25-165-s001.doc]

**SUPPLEMENTARY FIGURE LEGEND**

**Figure S1**. Covers to simulate the minimally invasive approach. A) Upper ministernotomy; B) Lower ministernotomy; C) Right lateral minithoracotomy; D) Left lateral minithoracotomy.


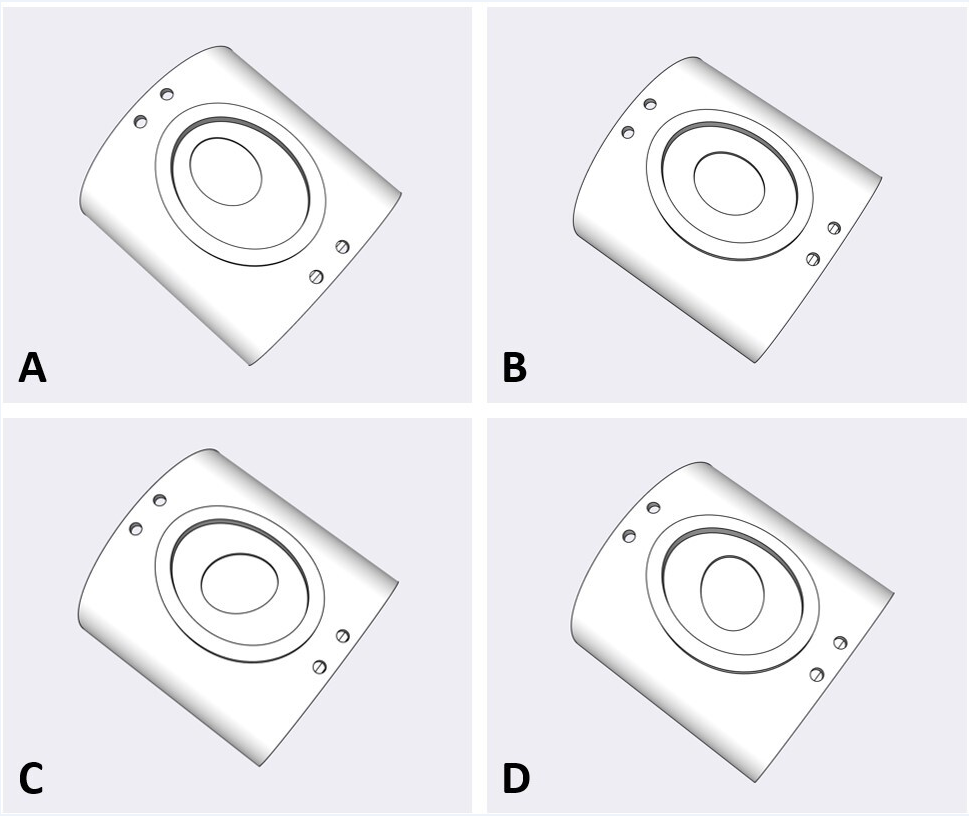


**SUPPLEMENTARY TABLE**

**Table S1**. Performance score evaluation of the surgical simulation on a model of aortic coarctation.

| **1. Tissue dissection (25 points maximum)** |
| --- |
| Has the isthmus been dissected? |
| Has the subclavian artery been dissected? |
| Has the proximal portion of the aortic arch been dissected? |
| Has the PDA been dissected? |
| Has the descending aorta been dissected? |
| **2. Transection of PDA (40 points maximum)** |
| Proximal ligation |
| Distal ligation |
| Was the proximal PDA suture at a safe distance from the left pulmonary artery (1-2 mm)? |
| Was the distal PDA suture at a safe distance from the aorta (1-2 mm)? |
| Was the PDA transection perpendicular to the vessel? |
| Was the transection clear (i.e., not jagged)? |
| Was there enough distance on both ends of the transected PDA? |
| Was the pulmonary portion reinforced with a stitch? |
| **3. Coarctectomy and preparation for the anastomosis (45 points maximum)** |
| Was the subclavian artery encircled? |
| Was the proximal clamp (aortic arch) positioned first? |
| Was the distal clamp (descending aorta) positioned later? |
| Was the proximal zone of the coarctation resected correctly (1-2 mm) from the PDA insertion? |
| Was the distal zone of the coarctation resected correctly (1-2 mm) from the PDA insertion? |
| Was the inner curvature of the arch incised? |
| Was the incision clear and sharp? |
| Was the incision extended into the arch (i.e., below the 2nd brachiocephalic vessel)? |
| Was the descending aorta incised correctly (on the left lateral side)? |
| **4. End-to-end Anastomosis (20 points maximum)** |
| Was the first stitch given on the distal aortic end? |
| Was the anastomosis started on the proximal end with a backward stitch? |
| Are all the sutures evenly spaced from one another with a gap of 1-2 mm between suture bites? |
| Are all the sutures an adequate distance from the edge (1-2 mm)? |

**VIDEO LEGEND**

**Video S1**. Simulation of aortic caorctectomy with end-to-end anastomosis on a 3D printed model by a senior surgeon.
